# Identifying and Optimizing Factors Influencing the Implementation of a Fast Healthcare Interoperability Resources Accelerator: Qualitative Study Using the Consolidated Framework for Implementation Research–Expert Recommendations for Implementing Change Approach

**DOI:** 10.2196/66421

**Published:** 2025-05-27

**Authors:** Jane Li, Emma Maddock, Michael Hosking, Kate Ebrill, Jeremy Sullivan, Kylynn Loi, Danielle Tavares-Rixon, Rajiv Jayasena, Grahame Grieve, Alana Delaforce

**Affiliations:** 1 Australian e-Health Research Centre CSIRO Brisbane Australia; 2 Australian Government Department of Health, Disability and Ageing Canberra Australia; 3 Health Intersections Pty Ltd Melbourne Australia

**Keywords:** Fast Healthcare Interoperability Resources accelerator, FHIR accelerator, Sparked, Consolidated Framework for Implementation Research, Expert Recommendations for Implementing Change, implementation enhancement plan, digital health, interoperability standards

## Abstract

**Background:**

Fragmented sharing of health information is known to negatively impact patient care and outcomes. To support the sharing of health information between systems, Fast Healthcare Interoperability Resources (FHIR) has emerged as the global interoperability standard for health information exchange. To speed up the process of adoption, various FHIR accelerator groups have been formed. FHIR accelerators such as the Sparked program in Australia enable communities and collaborative groups to develop high-quality FHIR standards for health care information exchange and encourage widespread uptake. However, limited research exists on the development, delivery, and implementation of FHIR accelerator programs.

**Objective:**

This study used qualitative methods to identify the key components of the Sparked FHIR accelerator, what factors influence implementation, and which strategies may help enhance its delivery.

**Methods:**

Semistructured interviews were conducted with Sparked stakeholders in the early stage of the program. The Sparked FHIR accelerator intervention components were described using a standardized reporting checklist (Template for Intervention Description and Replication). The Consolidated Framework for Implementation Research (CFIR) 2.0 was used to analyze factors influencing implementation. On the basis of a cumulative majority analysis, the most mentioned factors influencing implementation were identified. These factors were then mapped to the Expert Recommendations for Implementing Change (ERIC) tool to identify strategies for enhancing the implementation of the Sparked program.

**Results:**

A total of 17 participants were interviewed, including program leads, cochairs, representatives of software industry implementers, clinicians, and consumers. In total, 8 key CFIR influencing factors were identified: engaging, innovation design, assessing needs, local conditions, access to knowledge and information, partnerships and connections, capability, and work infrastructure. After mapping the top CFIR influencing factors to the ERIC tool, 5 strategy clusters were identified: adapt and tailor to context, develop stakeholder interrelations, support participants, train and educate stakeholders, and use evaluative and iterative strategies.

**Conclusions:**

This study enabled the core components of the Sparked FHIR accelerator to be defined and identified the factors that have the strongest influence on program implementation. Using the CFIR-ERIC approach facilitated the generation of expert-informed recommendations for improving the implementation of Sparked, but researcher recommendations were needed to supplement the tool. This research offers valuable insights for decision makers and implementers.

## Introduction

### Background

The adoption of digital health information systems and interventions is on the rise. However, the data informing these systems, such as requests for pathology tests and medical imaging, are often fragmented and represented using inconsistent terminologies and measurement units across various platforms. This phenomenon is known as the absence of interoperability. A lack of interoperability between digital health information systems and interventions can lead to adverse health outcomes and higher costs to the health system (eg, redundant laboratory testing, higher health care expenditure, and medication errors) [1-3]. To address fragmentation and promote interoperability, it is necessary to establish standardized measurements and terminologies that can be embedded into various health information systems. The Fast Healthcare Interoperability Resources (FHIR) [4] has rapidly emerged as one of the most favored standards for health information sharing and continues to show promise in developing an application-based approach to interoperability and health information exchange [5,6].

Health Level Seven (HL7) International, the global accredited standard development organization, has suggested 5 aspects of a rigorous standard development process that emphasizes consensus, fitness for purpose, implementability, community engagement, and ongoing maintenance [7]. During standard development, working groups adhere to a structured decision-making process to achieve consensus, openness, and fair representation of interests. In addition, all proposed standards undergo a formal balloting process to collect and reconcile feedback on specifications before they are published as normative standards. Despite the known benefits of implementing FHIR, work is needed to increase their uptake, adoption, and implementation [8,9].

Worldwide, numerous FHIR accelerators have been established to facilitate the development and adoption of agreed-upon core datasets, including Argonaut, CodeX, Gravity, and Da Vinci, as listed by HL7 [10]. These accelerators aim to develop local standards that expedite the adaptation and development of health information systems in an interoperable manner, enabling clinical information systems and health data applications to seamlessly and consistently share information regardless of the application source.

The Sparked accelerator program commenced in Australia in July 2023 [11]. This was part of the federal budget to implement new initiatives that improve digital health information sharing. It is facilitated by the Commonwealth Scientific and Industrial Research Organisation (CSIRO) Australian e-Health Research Centre and serves as a collaborative consortium to bring together government entities, technology vendors, health care organizations, peak bodies, practitioners, and domain experts to accelerate the creation, use, and adoption of national FHIR standards for health care information exchange [11,12]. By fostering community collaboration, Sparked aims to enable the community to develop robust, nationally agreed-upon, Australian context–specific HL7 FHIR standards, clinical information models, and terminology value sets. Technical and clinical design groups have been formed to bring a holistic perspective when defining requirements and specifications, as well as validating and implementing critical health care data and FHIR standards.

Although FHIR accelerators, including Sparked, have been carried out in various countries over the last few years, there is currently limited research available in peer-reviewed literature that reports on the development, delivery, implementation, and evaluation of accelerator programs. Furthermore, there is a lack of evidence concerning the key attributes and factors influencing the implementation of successful community practices in accelerator programs. A lack of formal intervention or program descriptions is not uncommon in the literature, and related work has focused on the technical development of FHIR implementation guides and pilot-testing of the FHIR standards to support a specialized domain, such as genomics, oncology, and health research [8,9,13-16]. Lessons learned from these smaller-scale individual developments have highlighted the importance of engagement from clinical work groups in addition to communication and coordination between the standard developers and the implementation team. These pilots have pointed out future directions in supporting community-driven HL7 FHIR accelerators, including the critical need for collaboration among the communities in developing consensus to accelerate interoperable data modeling and applications [9,13,14,16]. These lessons learned are often published on informal websites, and there is a lack of peer-reviewed evidence that operationalizes theory to help synthesize these learnings into generic recommendations. Standardized analysis and reporting of success can be achieved using implementation science.

Implementation science offers theories, models, and frameworks that help explain the conditions needed to optimize the uptake of evidence-based practice. In this context, there is evidence of the need to implement interoperable systems to promote health outcomes. In our study, the Consolidated Framework for Implementation Research (CFIR), a conceptual framework designed to systematically assess implementation using multiple lenses (called domains), was used to identify factors influencing intervention implementation [17,18]. The CFIR, resulting from a systematic review of available implementation frameworks, integrates factors associated with effective implementation from various theoretical and empirical studies into a set of domains and constructs. The CFIR comprises 5 major domains: intervention characteristics, inner setting, outer setting, characteristics of the individuals involved, and implementation process. Among these domains are 48 constructs and 19 subconstructs. It has been widely cited since its publication in 2009 and recently updated as the CFIR 2.0 [17-20]. The study authors developed a virtual care *expansion pack* to the CFIR 2.0 that includes extended constructs related to the uptake of virtual care innovations, and it used in this study [21]. On its own, the CFIR is highly useful for gathering insights into implementation, but to truly maximize the output data, it should be used in conjunction with an implementation strategy selection tool.

The Expert Recommendations for Implementing Change (ERIC) tool was first developed to create a refined taxonomy of implementation strategies that can be used to improve the uptake of evidence [22]. In 2019, the strategies were mapped to the CFIR framework to identify which strategies are best suited to addressing factors influencing implementation [23]. The mapping process occurred through consensus among implementation experts and resulted in the development of the CFIR-ERIC Matching Tool [24]. Despite known heterogeneity in agreement among experts as to what strategies best address CFIR constructs, approaches using the combined CFIR-ERIC tool have been demonstrated to improve the uptake of interventions, including one study that showed a 10-fold improvement in compliance with clinical recommendations related to the optimization of patients’ blood preoperatively [22,24-26]. The CFIR-ERIC approach can be flexibly applied and is particularly useful for guiding the evaluation of complex digital health care interventions, enabling the systematic identification of factors influencing the implementation of interventions across different contexts [27-30].

Implementation science promotes standardized reporting of interventions, and the Template for Intervention Description and Replication (TIDieR) checklist has been used to support clear articulation of intervention components [31]. Even when the optimal conditions for implementation are known, efforts to replicate these can be hampered by variations in the interventions for which the conditions are relevant. While we may understand the optimal conditions for implementing an FHIR accelerator, without knowing all the program elements that are necessary for success, key activities may be missed in future implementation attempts. For example, one FHIR accelerator may heavily rely on a mix of face-to-face and hybrid participation, and if this is not sufficiently articulated, other FHIR accelerators that instead rely on delivering the program entirely on the web may fail without realizing why despite having optimized the conditions for implementation. In this study, the TIDieR checklist was completed to enable replication of the Sparked program at the intervention level.

In this paper, we describe the core components of the Sparked FHIR accelerator and demonstrate how the CFIR-ERIC approach facilitated an understanding of factors influencing the implementation of the Sparked program. Our research provides actionable findings for decision makers and program implementers.

### Objectives

This study had three key objectives: (1) to define the core components of the Sparked accelerator program and understand how those components interact with each other, (2) to identify factors influencing the implementation of the Sparked accelerator program as perceived by relevant stakeholders, and (3) to leverage implementation science theory to develop an implementation enhancement plan to improve the adoption of FHIR standards by optimizing the Sparked FHIR accelerator program.

## Methods

Semistructured interviews were conducted with stakeholders of the Sparked program. Interview questions were developed based on CFIR constructs and focused on stakeholders’ perspectives on the program’s components, engagement experience, perceptions of the FHIR development process, and factors influencing its success.

### Participants and Recruitment

Participants included those involved in the conceptualization, coordination, and delivery of the Sparked program and its target population, such as program leaders, cochairs, members of the clinical and technical work groups, and consumer representatives. We aimed to recruit a minimum of 2 to 3 individuals from all relevant stakeholder groups to ensure adequate representation.

A Sparked program manager sent invitation emails to potential participants. A purposive, snowball sampling approach was used for recruitment, where additional stakeholders may be revealed as the research progresses [32]. Participants were provided with an electronic invitation (a recruitment flyer) that contained a link to complete a digital information and consent form through a secure REDCap (Research Electronic Data Capture; Vanderbilt University) server hosted by the CSIRO.

### Data Collection

Interview sessions were conducted over a 3-month period, with the first interview taking place 4 months after the program commenced. Each session was conducted via videoconferencing (Microsoft Teams; Microsoft Corp) and lasted 45 to 60 minutes. Audiovisual recordings of the interviews were professionally transcribed. In total, 4 authors conducted the interviews as available (JL, AD, EM, and RJ).

### Data Analysis

The transcribed data were exported into Microsoft Excel (Microsoft Corp) for cleaning and preparation for coding. Once complete, 3 researchers (JL, AD, and EM) carried out the coding process using the CFIR. A directed content analysis approach was adopted, enabling results to be aligned with CFIR 2.0 constructs. Additional constructs relevant to virtual care generated from a previous systematic review and designed as an extension to the CFIR, which are available as a preprint, were also used [21].

The initial coding was conducted independently by 2 researchers (JL and EM), and results were discussed and refined. Following this, a codebook was developed by 2 researchers (AD and JL) to define the CFIR constructs in the Sparked context (Multimedia Appendix 1). On the basis of the codebook, 2 researchers (AD and JL) independently evaluated and revised the initial results in a second-round coding and consistency-checking process. After finalizing the coding, a prioritization exercise was undertaken to identify the most mentioned influencing factors (barriers and enablers) based on the cumulative majority, similarly to other studies using the CFIR [27,28]. A list of brief statements for each of these factors was generated by the 2 researchers. Mapping of the barriers and enablers to the ERIC tool was conducted to select theory-informed strategies that could enhance the running of the Sparked program.

We reviewed information published on the Sparked website [9] and analyzed participants’ descriptions of Sparked components using the TIDieR checklist. We focused on the statements from program leaders and cochairs as they had an overall understanding of the program. The TIDieR items were further validated through author consensus (KE, JS, MH, KL, DT-R, and GG). The checklist enabled standardized reporting of Sparked components from an intervention perspective. Understanding the processes and contextual factors surrounding the Sparked program supported the development of the codebook during the analysis of CFIR influencing factors.

### Ethical Considerations

Ethics approval was obtained from the CSIRO Health and Medical Human Research Ethics Committee (project ID 2023_057_LR). All participants provided digital informed consent through the secure REDCap server hosted by the CSIRO before participation. Interview transcripts were deidentified before analysis, and the results were reported in an aggregated format to ensure participant anonymity. Participants did not receive compensation for taking part.

## Results

### Demographics of the Participants

A total of 17 participants were interviewed, including 5 (29%) program leads from the CSIRO, 4 (24%) cochairs, 3 (18%) technical design group members from software vendors, 4 (24%) clinical design group members, and 1 (6%) consumer representative. The demographics of the participants are summarized in Table 1. Participants’ digital health experience ranged from 2 to >25 years. Regarding HL7 and FHIR standards, their individual experiences varied from 1 to >25 years.

**Table 1:** Demographics of the participants (N=17).

Characteristic		Participants, n (%)
**Role**		
	CSIRO^a^ program lead or manager	5 (29)
	Cochair and lead	4 (24)
	Software vendor	3 (18)
	Clinician	4 (24)
	Consumer representative	1 (6)
**Sex**		
	Female	5 (29)
	Male	12 (71)
**Experience with digital health (years)**		
	>25	4 (24)
	15-25	7 (41)
	5-15	5 (29)
	<5	1 (6)
**Experience with standards (years)**		
	>25	3 (18)
	15-25	4 (24)
	5-15	4 (24)
	<5	6 (35)

^a^CSIRO: Commonwealth Scientific and Industrial Research Organisation.

### Sparked Components in the TIDieR Checklist

**Figure 1:**
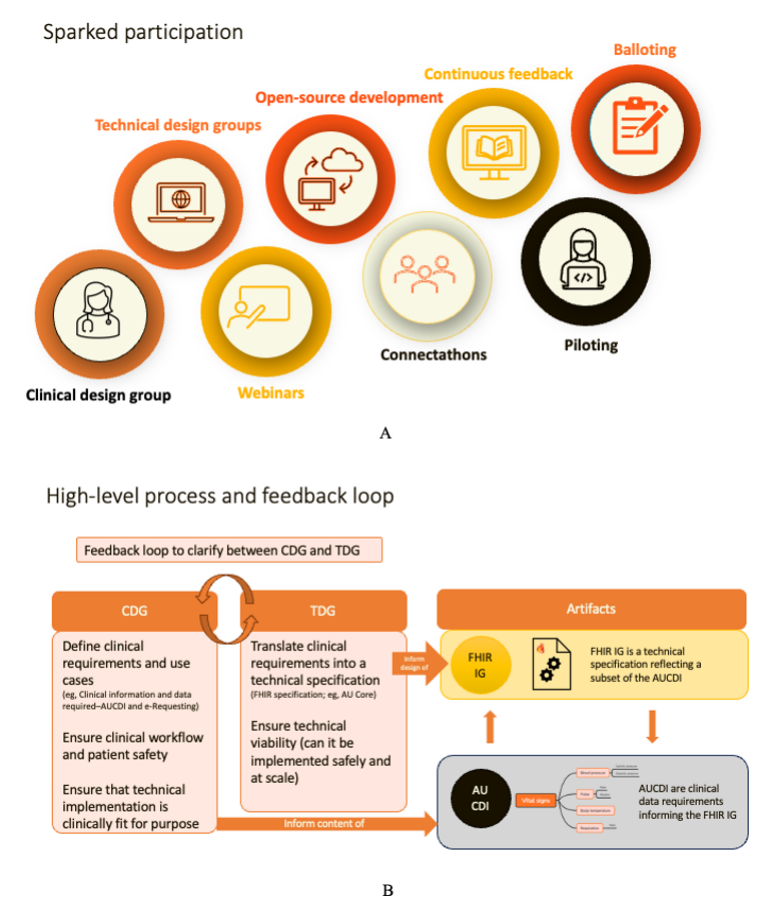
Sparked participation (A) and process of interaction and feedback loop (B). AU: Australian; AUCDI: Australian Core Data for Interoperability; CDG: clinical design group; FHIR: Fast Healthcare Interoperability Resources; IG: implementation guide; TDG: technical design group.

A detailed description of the Sparked intervention components is presented using the TIDieR checklist in Multimedia Appendix 2, including items and explanations related to the rationale, procedures, and materials; who was involved; and how the program is tailored, modified, and delivered. Figure 1 illustrates the components related to participation of design groups and their procedures and interactions with knowledge and artifacts.

### Top CFIR Influencing Factors

Using a cumulative majority (frequency of mention on a per-participant basis), our results identified factors that positively or negatively influenced the participants’ experience in the Sparked program and their perceptions of Sparked delivery.

A total of 65% (44/68) of the CFIR constructs were identified as factors influencing implementation. In addition, 44% (4/9) of the newly proposed virtual care CFIR constructs [21] were identified as being relevant. We then identified the top 25% most mentioned barriers and enablers and combined them to create a final list of 8 influencing factors to be addressed, as summarized and highlighted in Table 2. These 8 constructs were considered as key influencing factors and were selected for prioritization and ERIC mapping. These constructs were engaging, innovation design, assessing needs, local conditions, access to knowledge and information, partnerships and connections, capability, and work infrastructure.

**Table 2:** Top Consolidated Framework for Implementation Research influencing factors (N=17).

Construct	Participants, n (%)
Engaging	17 (100)
Innovation design	17 (100)
Assessing needs	16 (94)
Local conditions	16 (94)
Access to knowledge and information	15 (88)
Partnerships and connections	15 (88)
Capability	13 (76)
Work infrastructure	11 (65)

### Detailed Findings on the Top Factors Influencing Implementation

#### Overview

In the following sections, we present findings related to the 8 influencing factors organized by their CFIR domains. Their associated key (most mentioned) implementation condition statements in the Sparked context and exemplar quotes related to these statements are presented below. A full list of statements associated with each of these constructs is provided in Multimedia Appendix 3.

#### Innovation

*Innovation design* is related to how Sparked is designed and packaged, including how it is assembled, bundled, and presented. The key implementation condition statements for this influencing factor are presented in Textbox 1. Participants agreed that successful FHIR accelerators would require consensus-driven approaches, concurrent technical and clinical development, and effective coordination to ensure scalability and sustainability, supported by engagement that would encourage user interests and participation. Establishing a diverse community comprising the government, technology vendors, health care provider organizations, and experts was considered essential, and a centralized knowledge management system would be vital for information sharing among stakeholders.

**Innovation design—implementation condition statements.**

**A community comprising different stakeholders accelerates the creation and use of national Fast Healthcare Interoperability Resources standards**

- “The aim for Sparked is actually to build community...and build a really active community that together will actually localize and profile the FHIR Foundation, SNOMED etc for use within Australia. So it’s about coming together as a community together we go faster and actually fast tracking the adoption of these standards.” [P5]

**A facility to allow for concurrent technical and clinical development with opportunities to collaborate is needed**

- “The trouble part is the connection between the clinical and the technical, we all come from different camps and we all have different...agendas or approaches, what we technically think we are capable of doing against what they clinically really want...so there can be a lot of tension between the two and I would highlight...we just sort of don’t easily gravitate, integrate with each other.” [P11]

**A consensus-driven approach is a foundational component**

- “I think keeping a genuine focus on community and consensus like genuine open consensus, so not sort of closing anyone out and focusing on an approach that does enable sort of an open market and really using this to sort of set foundations for Australia and I think key to success is...trying to see this as a bipartisan, multi-year initiative.” [P15]

**A centralized knowledge management system is integral for information sharing**

- “They’re the two elements, sort of the technical artifacts and the specifications and then the sort of the community process and making sure that that’s inclusive, open and transparent and traceable...I think the biggest challenge...is making sure that people understand what they are, what the scope of them are, how they should be used, who should be governing them, how they should be maintained.” [P4]

#### Outer Setting

*Local conditions* is related to the economic, environmental, political, and technological conditions that enable the outer setting to support the implementation and delivery of Sparked. The key implementation condition statements for this influencing factor are presented in Textbox 2. Australia has a mature digital ecosystem that recognizes interoperability as a key priority. However, participants also felt that some sectors of health care lagged in understanding the importance and implications of interoperability. The program’s success may be influenced by uncertain political landscapes, and there was a perceived need for stronger support from educational and training institutions to enable a larger workforce to deliver program outputs.

*Partnership and connections* is related to how well the Sparked program and its members are networked with external entities. The key implementation condition statements for this influencing factor are presented in Textbox 3. Participants pointed out that Sparked had drawn individuals and organizations into participating, and the ongoing commitment of these stakeholders is vital for the program’s sustainability. These partnerships not only strengthen the credibility and global recognition of the program but also facilitate the sharing of valuable lessons learned. However, there was a perceived gap between the technical and clinical realms, and this could pose a challenge to the program’s output viability.

**Local conditions—implementation condition statements.**

**The digital ecosystem (at the macrolevel) in Australia has reached a requisite level of maturity**

- “I think we’re at a juncture in time in Australia where many in the industry, particularly the leaders that really are looking to see initiatives like FHIR really move forward, because of the efficiencies that it does bring the vendor, there’s at least for the time being there’s a window of opportunity for us to really bring them on board and use principles of good faith to work collaboratively together.” [P14]

**Some areas of health care (at the meso level) lack awareness of interoperability and its impact**

- “There’s different levels of maturity or organizations in terms of their understanding and awareness of what this is and what this means and specifically what this means for them. Like the aged care sector is quite a bit behind in terms of understanding how this works and how it can enable improved care delivery and information sharing.” [P4]

**The Sparked program’s viability hinges on potentially turbulent political changes**

- “I feel confident that the program can deliver, where it is today. It’s probably other forces that are political of nature that are always probably the bigger risk points of budget changes and all that sort of stuff change...Hopefully that won’t happen.” [P15]

**Greater engagement and support from educational and training institutions**

- “The biggest pain point that we’ve got, I mean general lack of resources in the country...There’s a finite set of FHIR experts, so how do we do this in the most efficient way possible...there’s a longer term around how do we encourage across, like across universities...it’s not shortage of people doing IT, it’s shortage of people doing IT that want to go into healthcare.” [P5]

**Partnership and connections—implementation condition statements.**

**The partnerships with important stakeholders support the sharing of lessons learned**

- “There’s many FHIR accelerators that are hosted by HL7 international...and a lot of those have been actually running a very long time...so we have been in communication with those folks for at least three years, sort of seeing what they’re doing, learning how they’ve done things, what are their lessons learned.” [P2]

**The partnerships with important stakeholders strengthen the credibility and visibility of Sparked to be recognized as a world-class Fast Healthcare Interoperability Resources accelerator**

- “Looking at the role of HL 7 International in supporting, particularly if you’re wanting to use Sparked as an exemplar...the Sparked program could go international with the blessing and support and potentially additional resources from the international community.” [P14]

**There is a perceived disconnect between the technical and clinical worlds impeding the viability of the program outputs**

- “You’ve got the clinicians providing the desirability and then you have the technologists or the TDG group providing the viability aspect, but it seems like there’s a disconnect there, like they need to be working together more, those two teams need to be collaborating somehow and provide decision-making in tandem as opposed to like one to the other.” [P10]

#### Inner Setting

*Work infrastructure* is related to the organization of tasks and responsibilities within and between individuals and teams and general staffing levels to support functional performance in Sparked. The key implementation condition statements for this influencing factor are presented in Textbox 4. Participants stated that balancing the need for progress with participants’ time and energy constraints was challenging, especially in extensive consensus building and in an accelerated environment. To ensure smooth operations and effective event management, comprehensive administrative and logistical preparation was required. Some participants pointed out that enhanced visibility of key participating organizations such as HL7 International would be essential for increasing community engagement, and the program would also require tools provided by software developers for delivering the test service and reference implementation.

*Access to knowledge and information* is related to how stakeholders in Sparked can access guidance and training to support their participation in the program. The key implementation condition statements for this influencing factor are presented in Textbox 5. Participants agreed that the Sparked program had ensured open access to program information and encouraged active use of its Confluence page. The program could benefit from a basic education package for individuals such as clinicians seeking to enhance their FHIR skills and from providing more detailed and user-friendly prereading materials.

**Work infrastructure—implementation condition statements.**

**Difficulty of balancing the need for progress with the demands on participants’ time and energy**

- “There’s a lot of community good dialogue needed...There’s not one way to solve the challenge...So consensus building takes time...and we’re working in an accelerated environment...It’s a lot of demand on their energy and time...I would say one of our challenges going forward will be sustaining the energy of our community to see it through.” [P2]

**Comprehensive administrative and logistical preparation is key for ensuring a smooth and productive event**

- “Throwing more resources at the problem isn’t necessarily going to solve the problem, but I do think in the case of the events coordination that would be something that would be beneficial and even a design team, a dedicated design team to help build much more digestible resources.” [P4]

**Enhanced visibility of each participating organization would enhance engagement**

- “The role of HL 7...needs to be more visible. I know that they’re a volunteer organization, so I think that’s a change that could be very helpful and they should be more independent anyway. So they are the standards organization here, so I think their role needs to be a bit more, just a bit more forward in this...that as an optical suggestion is I think really important.” [P15]

**Need for software developers to provide tools to support delivering the test service and the reference implementation**

- “Resourcing...for delivering the test service and the reference implementation and for our support of the standards we need tooling, software, tooling, so we need software developers to provide tools.” [P1]

**Access to knowledge and information—implementation condition statements.**

**A basic education package or resources for consumers or people wanting upskilling would be helpful**

- “There’s been certainly attempts to explain the FHIR, but I do feel like we need to say go a little bit...it is a technical topic, so there’s no avoiding it, but how do you make that a little bit more accessible for some of the, particularly some of the clinicians involved.” [P15]

**Anyone can access information about the Sparked program**

- “We have a confluence page that CSIRO hosts that that information is available on—like our road map, our plans, the clinical group actually sets off that and for the technical groups they actually set off the HL7 pages. So all that is public, so anyone can make a judgment and share that communication.” [P2]

**Prereading materials could be more detailed or easier to understand**

- “Just noting that...a good amount of the audience aren’t technical FHIR experts. So a little bit more concise direction in this is what we’re asking you to consider for this part of the workshop, these are the things that we’d like you to think about and discuss. I think just a little bit more clarity with those could’ve been helpful on the day.” [P17]

#### Individuals

*Capability* is related to stakeholders’ interpersonal competence, knowledge, and skills to fulfill their roles to participate in and deliver the Sparked program. The key implementation condition statements for this influencing factor are presented in Textbox 6. Enhancing training and education across various skill levels using practical examples was highlighted as a way to facilitate broader understanding and more meaningful engagement. There is a need to support technical and clinical knowledge development and address the challenge of enabling equal participation among stakeholders with varying backgrounds and knowledge levels. In addition to leveraging previous experience in FHIR activities to enhance capability, scaling existing formal academic training partnerships was identified as a potential solution.

**Capability—implementation condition statements.**

**Previous experience, knowledge, and involvement in other Fast Healthcare Interoperability Resources activities improve capability**

- “I’m lucky in that I had some maybe it’s tangential experience with FHIR...so I have some benefit of several years of that being developed and production...whereas most the people on those phone calls they are not from vendors who have had that US experience and so it’s going to take them a longer time to understand what is going on?” [P6]

**Formal academic training partnerships exist—they need to be scaled**

- “In the short term, what we are trying to do is how do we build as much capacity as possible. So there’s a training program which the digital health agency funds HL7, CSIRO and Uni of Melbourne to pull together to actually train as many people around FHIR. The shortest-term fix that we’ve got is actually to ramp up the training.” [P5]

#### Implementation Process

*Assessing needs* is related to how well the Sparked program assesses the priorities, preferences, and needs of stakeholders participating to guide the delivery of the program. The key implementation condition statements for this influencing factor are presented in Textbox 7. Engaging diverse stakeholder perspectives was considered as important for understanding participant requirements, and tangible examples are necessary to assess community and clinician needs effectively. Participants pointed out that achieving consensus among stakeholders with diverse backgrounds can be challenging. Providing discussion options in documents before meetings could help facilitate decision-making, and active participation has been encouraged in Sparked consensus processes, such as contributing ideas on Confluence pages. Insufficient representation from specific stakeholder groups (eg, consumers and individuals from rural areas) could risk excluding their voices. Ensuring adequate representation of special interest groups could enhance efficiency and relevance of input.

*Engaging* is related to how Sparked attracts and encourages participation. The key implementation condition statements for this influencing factor are presented in Textbox 8. Engaging all relevant stakeholders to enable meaningful co-design and stakeholder integration was seen as crucial to the accelerator’s success. Study participants believed that running events had played an important role in attracting participants and generating excitement and that the Sparked team had created an environment that supported and championed the engagement culture. Participants argued that ensuring an inclusive environment that accommodates diverse participant backgrounds and knowledge levels remained a significant challenge, whereas communicating the purpose and benefits of FHIR accelerators in accessible ways would be key to building interest. Creating a cohesive media and communication strategy was also considered as important despite challenges due to administrative and resource constraints.

**Assessing needs—implementation condition statements.**

**Community engagement that prioritizes listening to and recording of diverse stakeholder perspectives**

- “There’s been a real mix of like the people who’ve been in Digital Health for a long time but...a lot of new clinicians coming and joining...lots of people from different areas. So we’ve got a bunch of like immunologists who’ve joined, ED clinicians—all of these new digitally health young clinicians—I think that’s really exciting to have new people to help carry that energy forward.” [P3]

**Achieving consensus among different stakeholders can be challenging at times**

- “It’s too hard, we can’t do it or the technical people go and design this perfect solution and then the clinicians are like but that doesn’t work for me. So for us it’s really important that we remain in lockstep and make sure like yes we may be a little bit asynchronous, but we need to always be aware of where each other are going and not go too far ahead or in two different directions.” [P3]

**Ensuring adequate stakeholder representation and expertise of special interest groups ensures valuable input**

- “As an example, we look at the Royal College of Australasian Pathologists. They are in the category of clinical and so we funnel them into the clinical design group for input and they are also very key to the E-requesting. So we make sure that there are also on the technical and functional input for the E-requesting FHIR engine, not necessarily the core though, because that one doesn’t apply to them.” [P1]

**Engaging—implementation condition statements.**

**Engaging all relevant stakeholders is seen as crucial**

- “Overall the accelerator program has done a really good job of bringing communities together with a very diverse range of stakeholders and clinicians...so I think they’re doing the best they can and really trying to be a very inclusive environment, very open...there’s a genuine attempt by Sparked to gain consensus on decisions.” [P7]

**Communicating the purpose and benefits of Fast Healthcare Interoperability Resources accelerators in a way anyone can understand builds interest in participation**

- “It needs to be technically based, that’s what it’s about, but to get clinician engagement you need them to understand what the potential benefits are and that’s that marrying of the technical...you know this has been done I think very well by the CSIRO in the past and...to get clinicians involved.” [P8]

**Ensuring meaningful co-design and integration of stakeholder input**

- “It’s probably, it’s the best I’ve seen, an approach, of having a community approach, where everyone’s genuinely involved, rather than being told this is what we’re doing, we’ll have your feedback, but this is where we’re going and I do think it’s got a very good, so far, co-design culture about it and approach.” [P15]

**A dedicated effort to create cohesive media and communication strategies among partner organizations**

- “I understand it can be a nightmare managing so many people and so many things at the same time, but it’s just we’re getting a lot of emails...So far it’s been good...but sooner or later I’ll reach a stage where I’ll lag behind on all of these things...so how do we ensure we’re not spamming everyone with comms...it has to be very relevant to the point and ensuring engagement.” [P4]

### ERIC Strategy Mapping

The top 8 influencing factors were mapped to the ERIC tool to guide implementation strategy selection. We used the updated CFIR-ERIC Matching Tool [33] for this purpose. Strategies were subsequently categorized into clusters to make application easier [25]. Strategies that had the highest percentage of agreement (eg, highest expert consensus regarding being effective to address barriers or amplify enablers) were selected and generated into high-level recommendations. However, not all CFIR 2.0 constructs currently have recommendations. Where recommendations were absent, the research team reviewed the strategy list in conjunction with evidence to recommend strategies for use.

These strategies were collated into an implementation enhancement plan for action. The recommended ERIC strategy clusters include adapt and tailor to context, develop stakeholder interrelations, support participants, train and educate stakeholders, and use evaluative and iterative strategies, as outlined in Multimedia Appendix 4.

## Discussion

### Principal Findings: Identification of Influencing Factors for the Implementation of the Sparked FHIR Accelerator and Strategies for Enhancement

#### Overview

This study enabled the identification of the core components of the Sparked FHIR accelerator and the factors that positively and negatively influence program implementation. The CFIR supported the identification of key factors influencing the implementation of the Sparked program. Subsequent mapping to the ERIC framework enabled the development of tailored strategies for enhancing the implementation of FHIR accelerator programs. However, researcher recommendations accounted for >50% of the strategies due to a lack of consensus in the ERIC tool, suggesting that future work is needed to improve the reliability and comprehensiveness of the tool.

In this section, we examine the top influencing factors and associated recommendations generated through ERIC and researcher recommendations in the context of the current body of evidence.

#### Innovation Design

Innovation design was identified as a key enabler for Sparked in this study. Our results highlighted the core components required for a successful FHIR accelerator, including a consensus-driven approach, concurrent technical and clinical development, effective coordination for scalability and sustainability, and engagement that encourages user interests and participation. The collaborative consortium aspect of the FHIR program can be conceptualized as a community of practice. Challenges faced by communities of practice have been reported in studies of health care programs that involve diverse groups working together toward a common goal to address specific problems [34-36]. Similarly to insights reported from FHIR standard developments [9,34], our participants noted that achieving consensus among stakeholders with diverse backgrounds and needs could be difficult at times during standard development. In the context of large-scale digital health initiatives such as the United Kingdom’s delivery of digital health and well-being programs, tensions between communities of practice and the need for delivery at pace and scale were reported [37,38].

In digital health, co-design methods are used in the process of ideation, validation, and deployment involving both digital and health experts with different needs [39]. In the context of domain-specific FHIR standard development, community-driven connectathons, balloting, and centralized knowledge management systems for accessing program information have been pivotal in supporting the process [9,15]. Open dialogue between the clinical and technical work groups helps in confirming comprehension, resolving discrepancies, and providing feedback on standard specifications [9]. The ERIC tool suggests adapting and tailoring approaches to context, such as identifying the ways in which Sparked can be tailored to meet local needs while retaining core elements of the program to preserve its integrity (eg, generating tailored educational materials to ensure wide engagement), creating a use case–focused group structure to help bring clarity.

#### Local Conditions

Studies on large-scale digital health implementation have shown various levels of factors influencing readiness, including macro (eg, market, infrastructure, and policy), meso (eg, organizational), and micro (eg, professional or public) levels [37,38,40]. In Australia, the digital ecosystem involving the government, vendors, professional organizations, and clinicians has attained a requisite level of maturity to support interoperability [11,12]. However, our study found that certain health care sectors at the meso level still lack a full understanding of the significance of interoperability. Concerns were also raised about the potential effects of an unpredictable political climate on the program and challenges associated with enhanced data sharing, such as liability and cost.

A longitudinal evaluation of a national digital health initiative highlighted the need for greater investment in infrastructure, guidelines for safe and transparent use, incentives for interoperability, and upskilling for professionals and the public [38]. These recommendations are consistent with the researcher-selected ERIC strategies, which include securing commitments from key partners (eg, governments, professional colleges, and vendors who provide support or incentive to participate), involving existing Sparked governance structures (eg, boards of directors, HL7, and medical boards) to address quality management needs, and enhancing support from educational and training institutions to build a larger workforce to deliver program outputs.

#### Partnership and Connections

Sparked has attracted a diverse range of individuals and organizations, with their continued involvement playing a key role in ensuring the program’s long-term sustainability [11]. Partnerships with HL7 International will enhance the program’s international visibility and foster the exchange of valuable lessons learned [10]. However, the perceived disconnect between the technical and clinical domains in Sparked was raised as one of the challenges.

Research on developing genomics data FHIR standards highlights the importance of involving potential adopters (eg, pathology testing laboratories) alongside technical developers during standard development and pilot-testing [9]. Evaluations of digital health innovations in the Australian context have also emphasized the need for ongoing partnerships between innovation implementers and a diverse range of innovation recipients (eg, health care professionals and consumers) [30,41]. In addition to these insights, the ERIC tool suggests strategies for building a coalition, such as continuing activities that attract stakeholders, as well as promoting network weaving, such as building on existing high-quality working relationships and networks to promote a shared vision.

#### Work Infrastructure

One of the most frequently reported barriers to collaboration in health care innovations is that community members such as clinicians often lack the time to participate and prioritize other commitments, such as service provision [34,35]. Our study found that this challenge was compounded by the difficulty of progressing while balancing the time and energy constraints of participants, especially in extensive consensus building among work group members. From an infrastructure and resource perspective, the program also required software developers for test service delivery and reference implementation, as well as a coordination team for comprehensive administration and logistical tasks.

Recent research on communities of practice in health care settings suggests that nonhierarchical collaborations between participating organizations, shared ownership of outputs, and a centralized leadership structure with rotating leaders can be effective [42,43]. Similarly, the need for an enhanced visibility of key participating organizations such as HL7 International to increase community engagement was identified in our study. These suggestions align with the researcher-selected ERIC strategies, which include developing partnerships with organizations that have the necessary resources to implement the program, shifting and revising roles among professionals managing the program, and redesigning job characteristics if needed.

#### Access to Knowledge and Information

Effective knowledge management and information sharing are crucial for innovations involving communities of practice [36,42,43]. The Sparked program uses knowledge management systems such as the online collaboration workspace Confluence as a central resource for program information and hosts community events to support ongoing knowledge development. Our study participants were positive about the open access to program information and the promotion of active engagement with Confluence and events. They also suggested that individuals such as clinicians seeking to enhance their FHIR skills would benefit from additional educational resources.

Research on communities of practice has reported similar effective methods for creating shared information spaces, such as learning hub platforms with website resources and asynchronous online discussion forums and periodic email updates with news and forum notifications [42,43]. Using live transcripts for groups with diverse skills and sharing meeting recordings to accommodate varying schedules also help members catch up [42]. The ERIC tool also suggests several strategies for training and educating stakeholders, including conducting educational meetings tailored to different groups; developing manuals and toolkits in ways that make it easier to learn about the program; providing supporting materials for clinicians to facilitate understanding of Sparked program products; and distributing educational materials through various channels, such as in-person sessions, mail, and email.

#### Capability

Stakeholders’ requisite capabilities to participate in and deliver the Sparked program, such as health domain knowledge for technical work group members and FHIR knowledge for clinical work group members, were identified as an area for improvement in our study. Previous FHIR development efforts have also shown the need to support technical and clinical knowledge development and address the challenge of enabling equal participation among stakeholders with varying backgrounds and expertise levels [9].

While studies of digital health implementation have identified health care professionals’ digital technology skills, abilities, and experience as one of the barriers [30,44,45], research on communities of practice has shown that community members can continuously advance their knowledge through ongoing learning and active engagement [36]. Enhancing cross-training and education for technical and clinical work groups and across various skill levels, along with practical real-world examples, will help facilitate broader understanding and more meaningful engagement, as suggested by the ERIC tool. In addition to leveraging previous experience in FHIR activities to enhance capability, scaling existing formal academic training partnerships was identified by the ERIC tool as a potential solution.

#### Assessing Needs

Community engagement that prioritizes listening to and recording diverse stakeholder perspectives (eg, clinical, consumer, and technical levels) emerged as an enabler in understanding stakeholder needs related to program operation and delivery. Encouraging active participation in consensus processes and collecting feedback during the ballot process has enabled stakeholders to voice their thoughts and raise issues freely. However, limited representation from certain groups, such as consumers or individuals from rural areas, might result in their perspectives being overlooked.

Studies on FHIR development have shown the importance of adequate representation and expertise within special interest groups to drive efficiency and ensure that stakeholders’ needs are collected [9,13,14]. In addition to Sparked’s current practices of ongoing monitoring of participation and engagement, researcher-selected ERIC strategies identified ways to enhance needs assessment activities. These include using evaluative and iterative methods (eg, surveys) to gather and analyze information on stakeholder needs for their participation in the program and developing an audit schedule to check that agreed-upon processes (eg, data sharing) are consistently followed.

#### Engaging

Engaging all relevant stakeholders to enable meaningful co-design and integration was considered as both an enabler and a barrier by our study participants. The Sparked program has engaged stakeholders through various activities with a focus on enhancing participation through both in-person and online events. However, the size and pace of some events make it difficult for participants with less experience in standards to contribute effectively. A stronger effort is needed to gather insights from clinicians and nonexperts.

Our study has shown that, to engage a wider range of organizations and clinicians in the program, the concept of standards needs to be communicated in a way that aligns with their interests. As observed in other community-based health care consortiums, effectively communicating the purpose and benefits of a program in accessible ways is one of the approaches to build interest [34,42]. Participants in our study suggested a dedicated effort to enhance the media and communication strategy, such as more targeted and coherent emails and media content. A study on a large-scale national digital health program also highlighted the importance of clear communication and branding in a multi-agent, heterogenous partnership model [37,38]. It also emphasized the need to engage smaller business partners, who often show less interest in co-design compared to large multinational companies. Echoing the recommendations from digital health implementations [30,37,38,46], the ERIC tool suggested strategies related to further development of stakeholder interrelations, such as identifying champions who dedicate themselves to supporting, marketing, and driving the implementation; involving consumers and other currently underrepresented sectors; and offering more flexibility and opportunities to facilitate their active engagement.

### Implementation Science Approaches for Future Evaluations of FHIR Accelerators

Implementation science theory and approaches have been increasingly used in developing an improved understanding of how digital health services and innovations can be designed, developed, and delivered [27-30,47]. To our knowledge, no studies have reported the use of a systematic approach in evaluating FHIR accelerator implementation, thereby limiting the understanding of influencing factors and systematic investigation of the program implementation process. Our study illuminates the applicability and benefit of using the CFIR to identify factors influencing implementation and generate a structured, evidence-based plan to enhance it further. The findings of this study have informed an actionable implementation enhancement plan that has been developed by the Sparked team and will be evaluated later in the year.

We believe that the approach outlined in this study holds broad applicability and encourages others to adopt the CFIR with the ERIC tool to enhance the efficient delivery of accelerator programs. As FHIR and similar accelerator initiatives continue to expand [10], using research approaches and implementation evaluations guided by the CFIR and ERIC frameworks can facilitate more effective translation of the aims and objectives of FHIR accelerators and provide meaningful comparisons across them. These implementation science frameworks provide a common language and systematic approach for researchers to comprehensively study the implementation of multicomponent interventions. This approach enables synthesis of findings across different settings, interventions, and studies, contributing to an evidence base for understanding implementation processes and developing strategies to support successful collaborative FHIR standard development.

### Limitations and Future Work

While this study identified factors influencing the FHIR accelerator program and theoretically informed enhancement plan, several limitations should be acknowledged. First, this study was conducted within an accelerator program based in Australia; the results should be interpreted within the context, such as government support and Australian health care settings. Second, this study was conducted during the first quarter of the 2-year program, whereas ongoing efforts to improve the program by the Sparked management team have continued since then. Third, it is worth noting that the theoretically informed strategies will need to be discussed and co-designed with key stakeholders of the accelerator program for the Sparked team to develop actionable enhancement plans. Finally, this research forms part of a broader evaluation of the Sparked program, where we will incorporate a mixed methods design and additional evaluation data, such as surveys at multiple time points, to enrich the insights and contributions of the findings.

### Conclusions

This paper demonstrated how we identified the factors influencing collaborative practices and engagement within a complex accelerator program tasked with developing FHIR standards. It outlines recommendations to address the factors influencing the implementation of the program. Our methodology involved using the TIDieR guidelines to understand the activities of the Sparked accelerator program, the CFIR to develop interpretive statements (assessing the key influencing factors), and the CFIR-ERIC Matching Tool in conjunction with researcher recommendations (informed by evidence) to derive an implementation enhancement plan relevant to activities within the Sparked program. These insights facilitated the identification of strategic improvements that program leaders can use to adapt and refine activities to better engage technical, clinical, and other stakeholders, thereby supporting the delivery and outcomes of the accelerator program.

## Appendix

Multimedia Appendix 1 Codebook for the data analysis.

Multimedia Appendix 2 Sparked descriptions based on the Template for Intervention Description and Replication checklist.

Multimedia Appendix 3 Key influencing factors and associated statements.

Multimedia Appendix 4 Implementation enhancement plan.

## Abbreviations

CFIR: Consolidated Framework for Implementation Research
CSIRO: Commonwealth Scientific and Industrial Research Organisation
ERIC: Expert Recommendations for Implementing Change
FHIR: Fast Healthcare Interoperability Resources
HL7: Health Level Seven
REDCap: Research Electronic Data Capture
TIDieR: Template for Intervention Description and Replication
