# Supplementary material for: Identifying and Optimizing Factors Influencing the Implementation of a Fast Healthcare Interoperability Resources Accelerator: Qualitative Study Using the Consolidated Framework for Implementation Research–Expert Recommendations for Implementing Change Approach
Source: JMIR Med Inform. 2025 May 27;13:e66421. doi: 10.2196/66421 (PMC12152436; doi:10.2196/66421)
Supplement: Multimedia Appendix 1 [file medinform_v13i1e66421_app1.pdf]

Multimedia Appendix 1

Codebook for the data analysis

CFIR Domains/Constructs	Descriptions based on Sparked context
**Innovation**	
Source	Who is responsible for Sparked development and delivery?. E.g., CSIRO, DOHAC, ADHA, HL7
Evidence base	Is there evidence to support Sparked?
Relative advantage	What about Sparked makes it better than other programs or nothing at all?
Adaptability	Can Sparked be easily modified?
Trialability	Can Sparked be piloted and then rolled back?
Complexity	How hard is it to navigate the Sparked program?
Design	How is Sparked packaged, presented – what activities make it a program?
Cost	What is the cost of Sparked (from a user pays perspective)?
**Outer Setting**	
Local attitudes	How do current sociocultural values influence the uptake of Sparked?
Local conditions	Economic, environmental, political, and technological conditions enable the outer setting to support the implementation of Sparked
Partnerships & Connections	How well connected are the people involved with Sparked?
Policies and laws	How do current policies and laws influence engagement with and uptake of Sparked?
Financing	Is there sufficient and sustainable funding from external entities to support Sparked?
External pressure	Are there pressures that motivate people to be involved or support Sparked?
**Inner Setting**	
Structural characteristics	How is work conducted and organised in Sparked? (Physical, IT, Work)?
Relational connections	What is the quality of relationships among people involved with Sparked?
Communications	What is the quality/frequency, clarity of communications internally?
Culture	How important are the needs of all participants and are these valued?
Tension for change	How and why the current situation needs to change?
Compatibility	How does Sparked fit within the workflows of CSIRO, DOHAC, etc?

Relative priority	How important is Sparked in relation to other initiatives at organisation level?
Incentive systems	What are the incentives in place to support the delivery of Sparked?
Mission alignment	How well is the mission of Sparked aligned to sponsoring organisations?
Available resources	Is there adequate funding, space, and materials to deliver to activities of Sparked?
Access to knowledge & information	Can people in the Sparked program access guidance and training about it?
Governance	Are there local, inner setting policies and procedures to support the program (e.g., quality assurance, accountability, transparency, audit trail, data security and sharing)?
**Individuals**	
Roles:<br>(High-level; Mid-level; Opinion) leaders, Implementation (facilitators; leads; team members) etc	What roles are necessary for success and what do they contribute?
Need	Is there some sort of survival deficit fulfilled by the Sparked program?
Capability	Does everyone have the requisite capability to participate in, and deliver the Sparked program?
Opportunity	Does everyone in Sparked have a chance to participate and what influences that?
Motivation	What motivates people to participate in Sparked?
**Implementation Process**	
Teaming	How well does everyone join together to implement – are there processes to support that and what are they?
Assessing needs	How well does Sparked assess the needs of people participating in regard to how it operates and is delivered?
Assessing context	How well does Sparked assess barriers and enablers to implementation and what mechanisms allow for this (if any)?
Planning	How well does Sparked identify roles, responsibilities and milestones?
Tailoring strategies	Does Sparked consider and choose implementation strategies to support Sparked?
Engaging	How does Sparked attract and encourage participation?
Doing	Is Sparked iteratively changed and improved as it is delivered?
Reflecting and evaluating	How and if Sparked collects and acts upon feedback?
Adapting	How and if Sparked modifies its approach for optimal fit into inner setting processes?
