# Supplementary material for: Identifying and Optimizing Factors Influencing the Implementation of a Fast Healthcare Interoperability Resources Accelerator: Qualitative Study Using the Consolidated Framework for Implementation Research–Expert Recommendations for Implementing Change Approach
Source: JMIR Med Inform. 2025 May 27;13:e66421. doi: 10.2196/66421 (PMC12152436; doi:10.2196/66421)
Supplement: Multimedia Appendix 2 [file medinform_v13i1e66421_app2.pdf]

Multimedia Appendix 2

Sparked descriptions based on TIDieR

Template for Intervention Description and Replication (TIDieR) checklist item	Explanation
**Brief name**	Australian FHIR Accelerator - Sparked
**Why - rationale**	The Sparked FHIR Accelerator has been established to accelerate better healthcare outcomes for healthcare consumers, improved clinical healthcare delivery experiences and improve health system performance through the creation and implementation of standardized data and FHIR specifications.<br><br>The Sparked FHIR Accelerator is a nationally government funded initiative to grow a standards development community comprising government, software technology vendors, healthcare provider organizations, peak bodies, healthcare practitioners, consumers and domain experts to accelerate the creation and use of national data and interoperability standards for improved health information exchange across the Australian healthcare ecosystem.<br>It aims to enable the advancement of Australia's broader digital health and connected care agenda by ensuring interoperability and improved data quality and information flow across the health system. Sparked represents a strategic approach to fostering innovation and collaboration in healthcare interoperability, supporting improved healthcare delivery through better data sharing and system integration.
**What – materials**	The program will be conducted in collaboration with the stakeholder community using the Australian FHIR Community Process (AFCP), governed by HL7 Australia. Deliverables from the Sparked accelerator include:<br>- the Australian Core Data Set for Interoperability (AUCDI) as a core clinical information model and data set that underpins multiple local and international healthcare use cases<br>- the Australian eRequesting Data Set for Interoperability (AUeReqDI), a clinical information model and data set supporting requirements for pathology and medical imaging requests<br>- HL7 AU Core FHIR Implementation Guide, a technical specification for health information system implementers to use for health information sharing between software systems<br>- HL7 AU eRequesting FHIR Implementation Guide,<br>- Pathology and Radiology FHIR Value Sets to support the implementation of AUeReqDI and AU eRequesting FHIR Implementation guide for eRequesting use cases

	- A standards development community comprising of clinical and technical representatives from across the sector including the operation of standards development working groups (Clinical Design Group and Technical Design Groups).
**What – procedures**	Sparked is run by CSIRO's Australian eHealth Research Centre, and exemplifies the collaborative consortium approach, incorporating the expertise of the Department of Health and Aged Care, the Australian Digital Health Agency, and HL7 Australia. This unified front emphasizes the crucial role of interoperability and fosters a wider community collaboration to build robust FHIR standards, clinical data and information models, and clinical terminology value sets.
**Who will provide the intervention**	Sparked is a community comprising government, technology vendors, provider organizations, peak bodies, practitioners, and domain experts to accelerate the creation and use of national FHIR standards in health care information exchange.<br>The Sparked Clinical Data Design Group, AU Core Technical Design Group and the AU eRequesting Technical Design Group (the Design Groups) bring together key stakeholders with expertise and experience in clinical practice, software development, FHIR implementation and those who have health industry and practice knowledge to work in partnership.
**How will the intervention be delivered**	The Design Groups are open for any interested FHIR developer or implementer, software vendor, clinician, or domain expert to participate in the design, development and validation of the national clinical data sets and information models and FHIR Standards.<br>The program will be run through an open, transparent, co-design, co-development approach with participants forming design groups.
**Where will the intervention be delivered**	The program will be delivered as a series of face-to-face and online working groups, meetings, conferences, networking events and workshops.
**When will the intervention be delivered and over what period of time**	Following two-year program, draft standards for trial use will be published.
**Will the intervention be tailored?**	The Sparked program team adopted an agile approach to identifying ways to improve the program rapidly and changes are made iteratively.
**Will modifications be made**	From the management level, the program management board regularly meets to discuss the plan, modifications, and enhancements.<br>From the technical perspective, feedback and comments are collected via the Confluence page for The Australian Core Data for Interoperability (AUCDI) Release 1 before publications.
**How will adherence and fidelity be assessed?**	Underpinning this program is the Australian FHIR Management Framework (AFMF).<br>The Technical Design Groups are technical subcommittees under the governance of the HL7 AU FHIR Working Group (FWG) which oversees and coordinates the technical efforts related to the

	development of HL7 FHIR standards in Australia. They comply with the governance principles and practices of the FWG.
**How will changes be made?**	From the management perspective, CSIRO, as the facilitator, manages and coordinates modifications of the implementation plan and execution.<br>For example, in Release 1, from the technical perspective, after the review period, CSIRO works with the technical design group to work through to classify and action the feedback received from reviewers. Once Release 1 has been updated, the outcome of the review is shared with the Clinical Design Group.
