# Supplementary material for: Identifying and Optimizing Factors Influencing the Implementation of a Fast Healthcare Interoperability Resources Accelerator: Qualitative Study Using the Consolidated Framework for Implementation Research–Expert Recommendations for Implementing Change Approach
Source: JMIR Med Inform. 2025 May 27;13:e66421. doi: 10.2196/66421 (PMC12152436; doi:10.2196/66421)
Supplement: Multimedia Appendix 3 [file medinform_v13i1e66421_app3.pdf]

Multimedia Appendix 3

Key influencing factors and associated statements

CFIR domains/Constructs	Barrier	Enabler	Statements (number of participants mentioned)
**Innovation**			
**Innovation Design**			***How is Sparked packaged, presented – what activities make it a program?***
		✓	A community comprising government, technology vendors, provider organisations, peak bodies, practitioners, and domain experts to accelerate the creation and use of national FHIR standards in health care information exchange (14)
	✓	✓	A facility to allow concurrent technical and clinical development, with opportunities to collaborate are needed to support successful FHIR accelerators (10)
		✓	A consensus driven approach is a foundational component of a successful FHIR accelerator (6)
	✓	✓	A centralised knowledge management system is integral for information sharing among all stakeholders (6)
	✓	✓	A group responsible for overall coordination of the community is needed (5)
		✓	FHIR accelerators should be set up with consideration for future scale and sustainability (4)
	✓	✓	Groups that are focused on a use case help bring pragmatism and clarity (4)
	✓	✓	Inclusion of orientation and training resources would be useful to ensure wide engagement (4)
		✓	Provision of a test environment supports development of the standards relevant to local contexts (3)
	✓	✓	The size of working groups can impact the ability of organisers to effectively facilitate meetings (3)
		✓	A range of modalities to engage in meetings helps to support user interest and participation (1)
**Outer Setting**			
**Local conditions**			***Economic, environmental, political, and technological conditions enable the outer setting to support the implementation of Sparked***

		✓	The digital ecosystem (including government, vendors, professional organisations and clinicians) in Australia has reached a requisite level of maturity and sees interoperability as an important priority (13)
	✓		The Sparked program viability hinges on potentially turbulent political changes (2)
	✓		Some areas of health care are behind in terms of their understanding and awareness of what interoperability is and the impact it can have (2)
	✓		Government red tape may impede the ability of the program to deliver its intended outputs on time (1)
	✓		Greater engagement and support from education and training institutes is needed to support a larger workforce to participate in, and help deliver the Sparked program outputs (1)
		✓	Individual stakeholder needs have been put aside for the greater good of the Sparked program expected outputs (1)
	✓		Some stakeholders remain hesitant about the potential implications of enhanced data sharing (e.g. liability) which is driven by the current economic climate (1)
	✓		The necessary tailoring and associated expense is seen as a potential commercial hinderance by vendors (1)
	✓		There is a perception that support and engagement at the state level could be stronger (1)
	✓		Reimbursement mechanisms for uploading information are not supportive of cohesive information sharing and uptake (1)
**Partnerships & Connections**			***How well connected is the Sparked program and its participants with external entities?***
		✓	The Sparked program team are connected to, and inclusive of a wide range of diverse and important external stakeholders (12)
		✓	The partnership with important stakeholders strengthens the credibility and visibility of the Sparked program to be recognised a world class example of a FHIR accelerator (4)
		✓	The partnerships with important stakeholders supports sharing of lessons learned (4)

	✓		There is a perceived disconnect between technical and clinical worlds impeding viability of the program outputs (2)
		✓	CSIRO has a reputation for leading initiatives that attract external people and organisations to participate (2)
		✓	The commitment from external stakeholders supports the likelihood of program sustainability (2)
**Inner Setting**			
**Work Infrastructure**			***How is work conducted and organised in Sparked?***
	✓		Difficulty of balancing the need for progress with the demands on participants' time and energy - consensus-building takes time, especially in an accelerated environment. (9)
	✓	✓	Comprehensive admin and logistics preparation is key for ensuring a smooth and productive event. (2)
	✓	✓	Enhanced visibility of each participating organisation would enhance engagement (1)
	✓		Need software developers to provide tools to support delivering the test service and the reference implementation (1)
	✓		Participants stretched their capacity to its limits - for additional training tasks (1)
		✓	This difference in funding and organization structure influences the partnership between the Department, CSIRO, and HL7 Australia in driving the Sparked program (1)
**Access to Knowledge & Information**			***Can people in the Sparked program access guidance and training about it?***
	✓	✓	A basic education package/resources for consumers/people wanting upskilling would be helpful (7)
		✓	Anyone can access information about the Sparked program (7)
	✓	✓	Pre-reading materials could be more detailed or easier to understand (5)
		✓	Formal academic training partnerships exist - need to be scaled (3)
		✓	There is a knowledge management system that is a 'one stop shop' for accessing program information (2)

	✓		The knowledge management system (Confluence page) could be made more user friendly (2)
	✓		A gap exists in the context of broader messaging and communication through state and territory peak consumer organisations (2)
		✓	Events are a keyway of sharing knowledge and information (1)
		✓	Everyone is encouraged to contribute to the knowledge management system (1)
		✓	Governance structures around the storage of commercially sensitive information are in place (1)
		✓	Providing quality materials for training and education is seen as important (1)
**Individuals**			
**Capability**			***Does everyone have the requisite capability to participate in, and deliver the Sparked program?***
	✓	✓	Additional training and education available at multiple levels of expertise that feature real world examples and use cases would help support wider understanding, more meaningful participation (11)
	✓	✓	Challenge of creating an environment or event that enables equal participation due to varying backgrounds and knowledge levels among participants (6)
		✓	Prior experience, knowledge and involvement in other FHIR activities improve capability (2)
	✓	✓	Formal academic training partnerships exist - need to be scaled (2)
	✓	✓	A facility to allow concurrent technical and clinical development, with opportunities to collaborate are needed to support successful FHIR accelerators (2)
	✓	✓	Sustainability of capability and training to be considered (1)
	✓		The knowledge management system (Confluence page) could be made more user friendly (1)
**Implementation Process**			
**Assessing Needs**			***How well does Sparked assess the needs of people participating in regard to how it operates and is delivered?***

		✓	Community engagement that prioritises listening to, and recording of diverse stakeholder perspectives (including clinical, consumer and technical levels) is crucial to assess the needs to accelerator participants (12)
	✓	✓	Achieving consensus among different stakeholders (different levels of experiences in a technical or clinical field) can be challenging at times (8)
	✓		Ensuring adequate stakeholder representation and expertise at special interest groups helps drive efficiencies and ensures input is provided where of most use (7)
	✓		Insufficient representation from particular stakeholder groups (e.g. consumers, rural areas) in a workshop or meeting, may result in the lack of the voice or preferences from those groups. (2)
	✓		A facility to allow concurrent technical and clinical development, with opportunities to collaborate are needed to support successful FHIR accelerators (2)
	✓	✓	Assessing the needs of community and clinician participants is hard without tangible examples (2)
		✓	Gathering participants' perspectives during the ballot process, allowing them the freedom to comment and raise any topic. (2)
		✓	Provide options in documents prior to meetings for discussion and decision making in meetings (2)
	✓		Participants need to actively participate in Sparked processes -e.g. putting ideas on Confluence pages to be heard (1)
**Engaging**			***How does Sparked attract and encourage participation?***
	✓	✓	Engaging all relevant stakeholders is seen as crucial to the success of the Sparked FHIR accelerator (15)
	✓	✓	Communicating the purpose and benefits of FHIR accelerators in a way that anyone can understand builds interest in participation (5)
		✓	Ensuring meaningful co-design and integration of stakeholder input is seen as important for maintaining engagement (5)
	✓		Challenge of creating an environment or event that enables equal participation due to varying backgrounds and knowledge levels among participants (4)
	✓		A dedicated effort to create a cohesive media and communications strategy among partner organisations is needed (4)

		✓	Promotion through LinkedIn and email as an adjunct to attracting people to Sparked (4)
	✓	✓	Multimodal options are attractive to people who are time poor (3)
	✓	✓	Need a business case to really attract clinical and professional technical folks (including overseas) to be involved in the discussions on a day-to-day basis (3)
	✓		Challenge of community engagement due to various difficulties (administrative, resource, effort required) (2)
	✓	✓	Engagement with peak consumer organisations would be beneficial (1)
		✓	Interest and investment by the government assists in attracting people to be involved with Sparked (1)
	✓		Need to engage with some stakeholders (e.g., smaller hospitals) who may lack the resources and motivations to adopt FHIR (1)
