# Supplementary material for: Identifying and Optimizing Factors Influencing the Implementation of a Fast Healthcare Interoperability Resources Accelerator: Qualitative Study Using the Consolidated Framework for Implementation Research–Expert Recommendations for Implementing Change Approach
Source: JMIR Med Inform. 2025 May 27;13:e66421. doi: 10.2196/66421 (PMC12152436; doi:10.2196/66421)
Supplement: Multimedia Appendix 4 [file medinform_v13i1e66421_app4.pdf]

Multimedia Appendix 4

Implementation enhancement plan

ERIC cluster (influencing factor)	ERIC recommendations (most strongly recommended)	Cumulative percent endorsement [33] or Researcher recommendation	Strategies tailored to the Sparked context
**Adapt and tailor to context (Innovation Design)**	Promote adaptability	48	**Identify the ways in which Sparked can be tailored to meet local needs and clarify which elements of the innovation must be maintained to preserve fidelity.**<br>E.g. identify changes that could be made to make meetings more efficient and more targeted.
**Develop stakeholder Interrelations (Local Conditions)**	Obtain formal commitments	Researcher recommendation	**Obtain written commitments from key partners that state what they will do to implement the Sparked program.**<br>E.g. Continue to seek written agreements from government, colleges, vendors etc. who provide support and/or are incentivized to participate to ensure a return on investment and promote accountability.
	Involve executive boards	Researcher recommendation	**Involve existing governing structures (e.g., boards of directors, medical staff boards of governance) in the implementation effort, including the review of data on implementation processes.**<br>E.g. Ensure that there are lines of communication to relevant boards and continue and expand these. Ensure provision of regular reports that may include items identified as suitable for audit or as necessary for monitoring the progress of the program which may be decided upon during the discussion of earlier recommendations regarding auditing and quality management system establishment.
**Develop stakeholder Interrelations (Engaging)**	Identify and prepare champions	63	**Identify and prepare individuals who dedicate themselves to supporting, marketing, and driving through an implementation, overcoming indifference or resistance that Sparked may provoke in an organization.**<br>E.g. If appropriate, and as strategically needed, recruit people or identify people as Sparked champions. Encourage them to promote the program.
	Involve consumers	Researcher recommendation	**Develop a suitable engagement plan for including consumers in the standards development process.**<br>E.g. Engage with state and national consumer groups to determine suitable consumer input into standards development.

	Prepare consumers to be active participants	Researcher recommendation	**Prepare consumers to be active, to ask questions, and specifically to inquire about the Sparked program, the evidence behind decisions etc.**<br>E.g. Provide consumer specific training
**Develop stakeholder Interrelations (Partnerships & Connections)**	Build a coalition	62	**Recruit and cultivate relationships with partners in the implementation effort.**<br>E.g. Continue and expand upon activities that attract people to participate in Sparked.
	Promote network weaving	50	**Identify and build on existing high quality working relationships and networks within and outside the organization, organizational units, teams, etc. to promote information sharing, collaborative problem-solving, and a shared vision/goal related to implementing the Sparked program.**<br>E.g. Continue to identify important stakeholders and opportunities to increase the overall network of those who are part of the Sparked program.
	Develop academic partnership	50	**Partner with a university or academic unit for the purposes of shared training and bringing research skills to an implementation project.**<br>E.g. Build upon existing university links and identify additional institutions that may be interested in developing tertiary courses.
**Supporting participants (Work Infrastructure)**	Develop resource sharing agreements	Researcher recommendation	**Develop partnerships with organizations that have resources needed to implement the Sparked program.**<br>E.g. Continue to foster and expand partnerships with organizations that can help support Sparked and it's longer-term goals.
	Revise professional roles	Researcher recommendation	**Shift and revise roles among professionals who run Sparked, and redesign job characteristics.**<br>E.g. Consider what roles are needed for Sparked to function and what the responsibilities are and have a central document that formally outlines this so that it is clear where accountability lies. This should extend to exploring who or what organizations are responsible for the longer-term goals of Sparked, including adoption of the standards.
**Train and educate stakeholders (Access to Knowledge & Information/ Capability)**	Conduct educational meetings	70	**Hold meetings targeted toward different stakeholder groups (e.g., providers, administrators, other organizational stakeholders, and community and consumers) to teach them about the Sparked program.**<br>E.g. Webinars, face-to-face meetings, and podcasts.
	Develop educational materials	59	**Develop and format manuals, toolkits, and other supporting materials in ways that make it easier for stakeholders to learn about the Sparked program and for clinicians to learn how to support the adoption of the Sparked program products.**<br>E.g. Digestible resources on the Sparked website that provide recipients with the necessary knowledge.

	Distribute educational materials	55	**Distribute educational materials (including guidelines, manuals and toolkits) in person, by mail, and/or electronically.**<br>E.g. Create a request function on the website for information or materials to be emailed as part of a sign-up process.
**Use evaluative and iterative Strategies (Assessing Needs)**	Audit and provide feedback	Researcher recommendation	**Monitor progress and adjust practices and implementation strategies to continuously improve the quality of Sparked program delivery.**<br>E.g. Develop a set of processes that one thinks other accelerators need to adopt to ensure the smooth running of the program and use this as a measurement tool to track progress towards goals or check intended processes.
	Develop and organize quality management system	Researcher recommendation	**Develop and organize systems and procedures that monitor processes and/or outcomes for the purpose of quality assurance and improvement.**<br>E.g. Develop an audit schedule to check that agreed internal processes are being followed (data sharing, contact lists).
	Conduct a local needs assessment	Researcher recommendation	**Collect and analyze data related to the need for the Sparked program (e.g. surveys).**<br>E.g. Distribute surveys over time to track changes in sentiment etc.
